# Sustained abstinence after a methamphetamine-specific treatment program for pregnant and parenting women

**DOI:** 10.1038/s41598-025-17808-2

**Published:** 2025-09-25

**Authors:** Maximilian Pilhatsch, Fabienne Körner, Maik Spreer, Arvid Pietsch, Alexa Fries, Johannes Petzold

**Affiliations:** 1 Department of Psychiatry and Psychotherapy, Carl Gustav Carus University Hospital, Faculty of Medicine, Dresden University of Technology, Dresden, Germany; 2 German Center for Child and Adolescent Health (DZKJ), Partner site Leipzig/Dresden, Dresden, Germany; 3 Department of Psychiatry and Psychotherapy, Klinikum Dresden (Friedrichstadt), Dresden, Germany

**Keywords:** Psychology, Health care

## Abstract

The treatment of methamphetamine use disorder (MUD) poses a significant challenge due to high rates of treatment discontinuation, substance use recurrence, and socioeconomic adversity. ‘Mummy, think of me’ (MAMADAM, an acronym from the German “Mama, denk an mich”), a treatment program specifically designed for pregnant women and young mothers with substance use disorders, has shown positive results regarding treatment efficacy and retention of child custody. This study aimed to evaluate the long-term sustainability of the MAMADAM intervention, which has not yet been examined. For this retrospective follow-up study, former patients with MUD who were pregnant and/or parenting women at the time of MAMADAM treatment were contacted by telephone. We analyzed whether self-reported abstinence after discharge from MAMADAM was associated with patient characteristics at admission to MAMADAM. Of the 114 eligible women, 39 participated in the follow-up interview, on average, 4.5 years after completing MAMADAM. Of these, 16 (41%) reported sustained abstinence from all addictive substances, excluding tobacco. At 12 and 18 months after discharge from MAMADAM, 9/39 (23%) and 11/39 (28%) women, respectively, reported at least one instance of substance use, including alcohol but excluding tobacco. Women who reported recurrences of substance use were significantly more likely to have more underage children, live apart from at least one minor, and consume methamphetamine for longer before MAMADAM than women with sustained abstinence. Cox regression, controlling for the duration of MAMADAM treatment, indicated that a longer history of methamphetamine use at admission to MAMADAM was associated with a shorter period of abstinence from all addictive substances (excluding tobacco) following discharge. The MAMADAM program is effective and sustainable in supporting long-term abstinence among pregnant and parenting women with MUD, indicating that pregnancy and parenthood can serve as turning points for promoting the necessary behavioral changes for recovery from addiction. Some women remain at elevated risk of relapse due to the vulnerabilities identified in this study. Future research should investigate whether stronger collaborations with government and community agencies can help these women maintain long-term abstinence.

## Introduction

Methamphetamine (MA) is regarded as one of the most significant illicit substances of the present era^1^. Among all substance use disorders (SUDs), methamphetamine use disorder (MUD) presents a substantial global challenge^2^. The high availability of MA in certain regions, its highly addictive pharmacological properties, the frequent occurrence of psychiatric and somatic comorbidities, and widespread psychosocial stress factors, such as homelessness and delinquency, further complicate the course and treatment of MUD^3^. Patients with these complex needs often face limited therapeutic resources and significant deficiencies in healthcare. This results in an unfavorable prognosis, characterized by prolonged and severe courses of illness, high rates of relapse, and, in many cases, fatal outcomes^3,4^.

This healthcare gap has a particularly adverse impact on young mothers with MUD and their children. In the U.S., 6.8% of young women reported lifetime MA use, which was associated with a 2.9-fold increased likelihood of experiencing pregnancy compared to those who had never used MA^5^. The pharmacological properties of MA have been linked to increased risk-taking behavior, heightened impulsivity, and, in some cases, enhanced sexual sensation^3,6,7^. This results in an increased number of unplanned pregnancies among persons who use MA compared to the general population^6^. Continued MA use during pregnancy has been demonstrated to have a detrimental impact on both mothers and their developing offspring, including fetal growth restrictions and reduced brain volume^8,9^. For the affected mothers, pregnancy, childbirth, and parenthood have traditionally been perceived as additional stressors that may further complicate SUD^10^. However, evidence from the interdisciplinary treatment program ‘Mummy, think of me’ (MAMADAM, an acronym from the German “Mama, denk an mich”)—established in 2016 at the University Hospital Dresden by the Departments of Psychiatry, Obstetrics, and Pediatrics—suggests that childbirth can serve as a critical turning point for behavioral change in addiction^10,11^.

Details of the program’s therapeutic modalities and treatment outcomes have been described elsewhere^9,12,13^. Briefly, pregnant women and young mothers with MUD are primarily treated on an outpatient basis, with the option of inpatient treatment if necessary. Treatment is provided in a multi-modal and multi-professional manner over an average duration of eight months. It includes transparent coordination with child welfare services. The therapeutic components have been subject to continuous optimization, such as the implementation of supportive abstinence monitoring and the integration of social services. Rapid access to individual psychotherapy has also been integrated to address psychiatric comorbidities^9,14^. As a result, the retention rate was as high as 68% by 2020^10,12^. Another major objective was the retention of child custody, ensuring that children could remain with their previously substance-using mothers, which was achieved in approximately 72% of cases^10^.

Despite these encouraging results, there is currently no evidence available regarding the long-term effectiveness of the MAMADAM program. Information on sustainability is particularly relevant, as recurrence of substance intake is a common phenomenon in SUDs, even years after cessation. Therefore, this study aimed to characterize the long-term course of patients following their participation in the MAMADAM program. A systematic follow-up survey was conducted to identify factors associated with the primary goal of abstaining from all addictive substances (excluding tobacco).

## Methods

### Data collection

In a previous study, we screened 118 women with MUD according to the Diagnostic and Statistical Manual of Mental Disorders, Fifth Edition (DSM-5). Sixteen women were excluded as they had still been in MAMADAM care at that time. The remaining 102 women were analyzed as they had either discontinued or completed MAMADAM care^13^. This cohort of 102 pregnant and/or parenting women included all 73 women whose MAMADAM treatment adherence had been previously analyzed^12^. For the present follow-up study of MAMADAM, 4 of the original 118 women were still in MAMADAM care, leaving 114 eligible participants.

A suitably trained research assistant who was not part of the MAMADAM care team attempted to call these 114 former patients up to 4 times over 169 days. The purpose was to invite them to participate in a standardized follow-up interview based on predefined questions from the German Addiction Treatment Statistics (Deutsche Suchthilfestatistik)^15^. Participants were excluded if they could not be reached by telephone or if they declined to participate. Thirty-nine former patients provided informed consent and completed the 15-minute phone interview. Abstinence rates were based on retrospective self-reports. Participants who reported that they had not been continuously abstinent since discharge from MAMADAM were asked to specify the substances they had consumed, using a standardized checklist of common addictive substances.

This study was approved by the Ethics Committee at Dresden University of Technology, Germany (BO ff (Mono)-EK-55022025). All research was performed in accordance with relevant guidelines/regulations and the Declaration of Helsinki. Informed consent was obtained from all participants.

### Statistical analyses

SPSS 29 (IBM, Armonk, NY, USA) was used for two-sided tests with a significance level of *p* < 0.05, including complete data on all patients unless missing values resulted in reduced sample sizes. Categorical variables were compared using Pearson’s chi-square test or Fisher’s exact test when the expected cell count was less than five. Continuous variables were compared using Mann-Whitney U tests, as histograms, normal quantile-quantile plots, and tests of normality demonstrated that continuous data were mostly not normally distributed.

To assess potential response bias, we compared sociodemographic and clinical data at admission to MAMADAM, as well as the duration and success of MAMADAM treatment between women with and without follow-up data. We categorized women with follow-up data into two groups based on whether they reported sustained abstinence (excluding tobacco) since MAMADAM discharge until follow-up. We calculated group comparisons of (1) sociodemographic and clinical characteristics at admission to MAMADAM, (2) the duration and success of MAMADAM treatment, and (3) time from MAMADAM discharge to follow-up.

We calculated Spearman’s correlations between the variables that differed significantly between the groups. These variables were the number of underage children, presence of mother-child separation, and years of MA use. All of these significant variables were significantly inter-correlated and thus shared considerable variability in our relatively small study sample. Therefore, only the variable with the strongest significance regarding group differences (i.e., years of MA use) was included in the Cox regression to test its predictive value on the duration of sustained abstinence since discharge from MAMADAM, controlling for the duration of MAMADAM.

## Results

### Characterization of study sample

Of 114 former patients with MUD, 39 (34%) participated in the follow-up interview, which was conducted, on average, 4.5 years after they completed the MAMADAM program. Interviewees were more often in stable abstinence at MAMADAM discharge than patients without follow-up data, without significantly differing in sociodemographic or clinical characteristics at MAMADAM admission or in the duration of MAMADAM treatment (Table 1).

### Likelihood and duration of abstinence

**Fig. 1:**
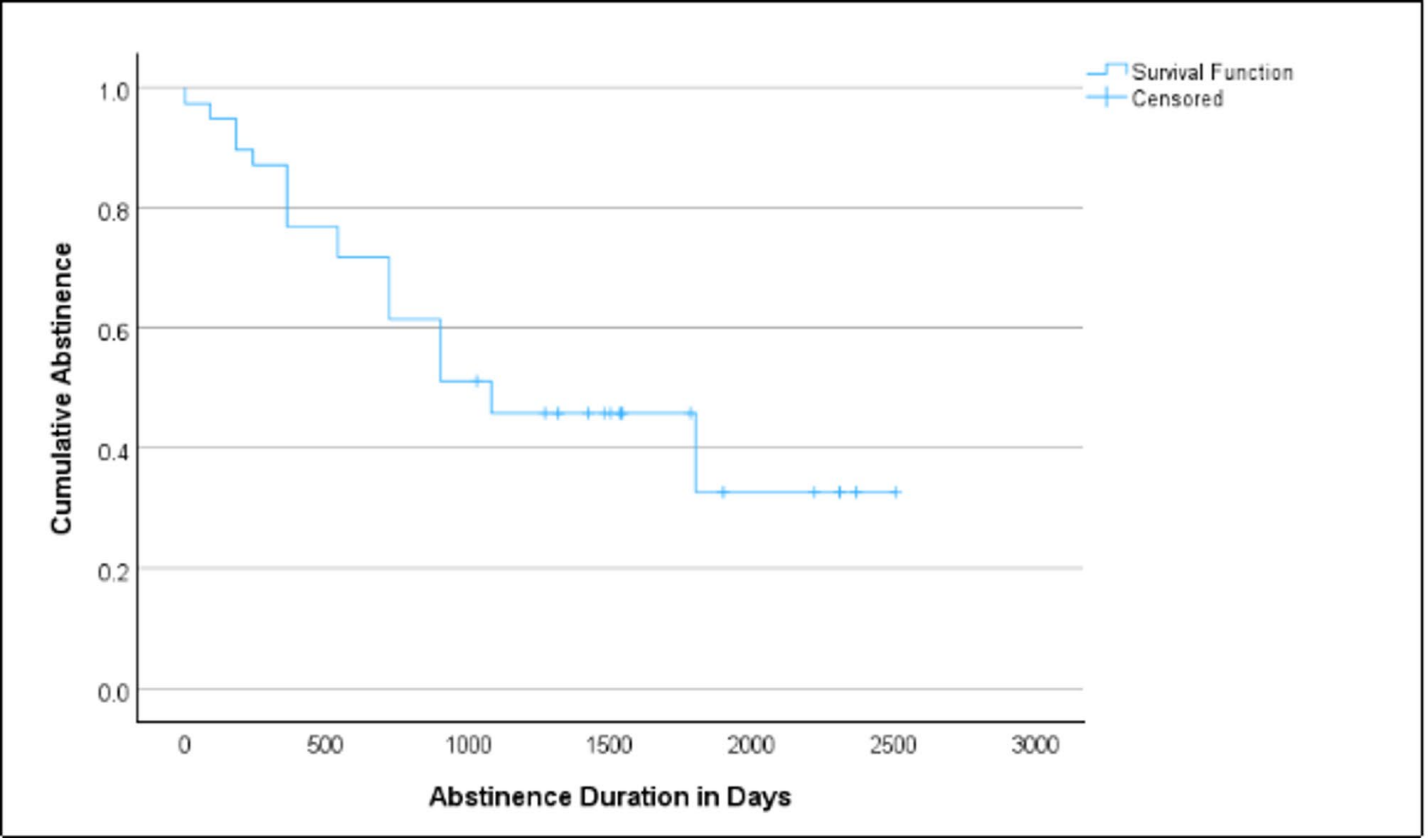
Kaplan-Meier estimator of abstinence duration after MAMADAM until follow-up *N* = 39.

Of the 39 participants, 16 (41%) reported that they had maintained abstinence from all addictive substances, excluding tobacco, throughout the entire follow-up period (mean ± SD: 1689 ± 444 days). Of these 16 women, 12 reported tobacco use during follow-up. The remaining 23 participants reported that they had used at least one addictive substance other than tobacco following an average abstinence period of 672 ± 476 days. Among these 23 women, the following substances were reported: MA (*n* = 14), cocaine (*n* = 3), stimulants other than MA or cocaine (*n* = 3), alcohol (*n* = 14), cannabis (*n* = 11), opioids (*n* = 1), and hallucinogens (*n* = 1). Consequently, 25 of 39 patients (64%) reported MA abstinence from MAMADAM discharge until follow-up. After 12 and 18 months following the discharge from MAMADAM, 30/39 (77%) and 28/39 (72%) patients, respectively, reported sustained abstinence from all addictive substances, excluding tobacco (Fig. 1).

### Potential correlates of sustained abstinence

Compared with women who maintained abstinence from all addictive substances, excluding tobacco, since discharge from MAMADAM, women who resumed use were more likely to have a higher number of minor children, live separated from at least one minor, and have a longer history of MA use at admission to MAMADAM (all p-values < 0.05, Table 2).

### Correlations between potential correlates of sustained abstinence

All three variables that differed significantly between women who reported sustained abstinence and those who reported using substances during the follow-up period were significantly and positively inter-correlated. Specifically, a longer history of MA use was associated with a higher number of underage children and more frequent mother-child separations (all p-values < 0.05, Table 3).

### Prediction of sustained abstinence

**Fig. 2:**
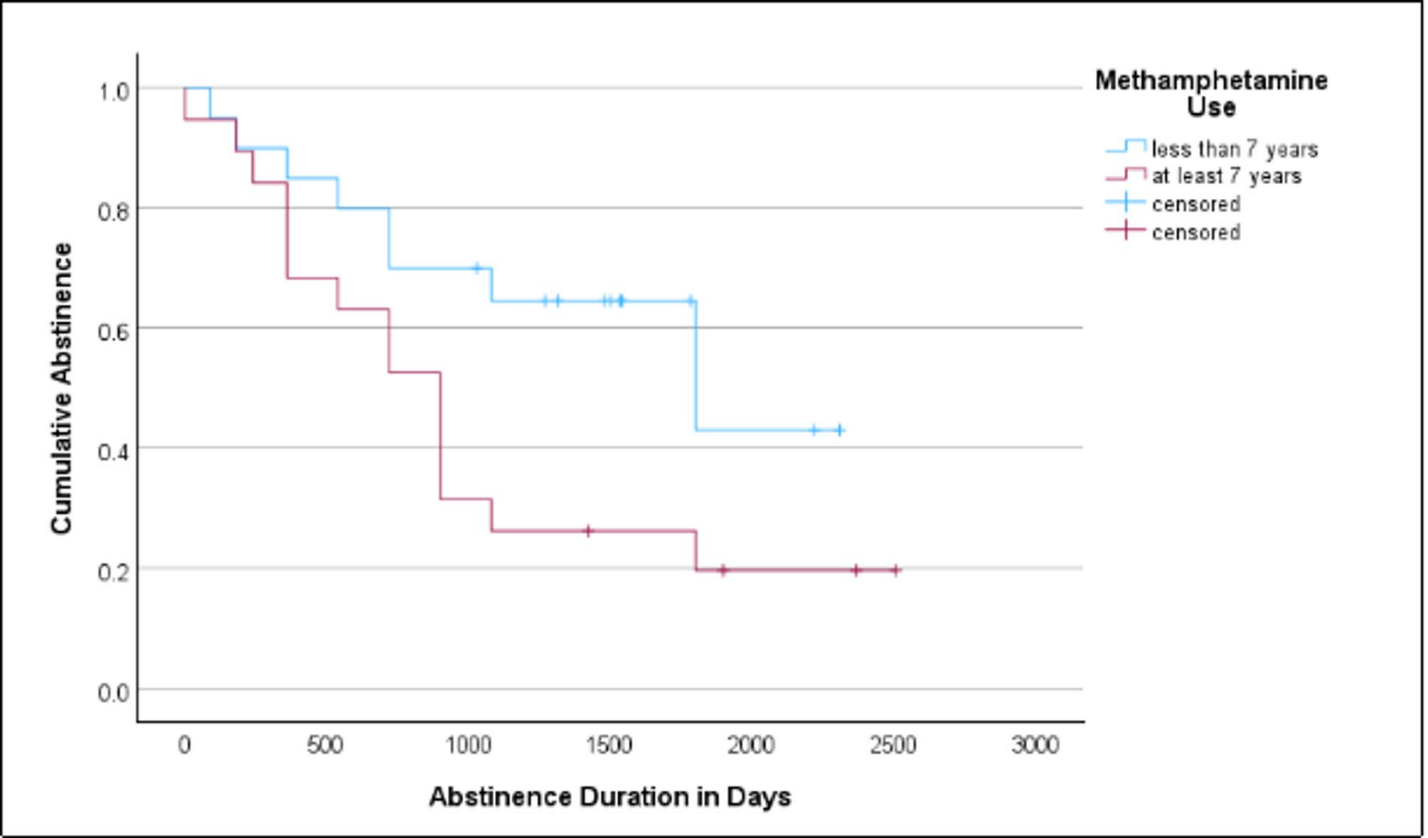
Kaplan-Meier estimators of abstinence duration after discharge from MAMADAM until follow-up, stratified by duration of methamphetamine use at admission to MAMADAM.

Cox regression analysis indicated that a longer history of MA use at admission to MAMADAM was associated with a shorter duration of abstinence from all addictive substances (including alcohol but excluding tobacco) after discharge from MAMADAM (Fig. 2). In contrast, the duration of MAMADAM treatment did not predict the duration of abstinence (Table 4). The duration of MA use before admission to MAMADAM was not significantly correlated with the duration of MAMADAM care (*p* = 0.69).

## Discussion

The findings of this study indicate that a close-meshed therapy program initiated during pregnancy or parenthood can have a sustained positive impact on the course of MUD. Almost 80% of the follow-up participants reported sustained abstinence from all addictive substances, except for tobacco, for at least one year, and more than 50% remained abstinent for 2.5 years. Only 36% of the follow-up participants reported any MA use throughout the follow-up period, which averaged 4.5 years.

In typical clinical populations of MA users who sought treatment voluntarily, the post-treatment recurrence rate has repeatedly been found to exceed 60% at the 12-month follow-up^16–18^. By contrast, Kamp et al. (2020) documented lower recurrence rates of 24% and 32% at 12 and 18 months, respectively, following a structured treatment program^19^. Huang et al. (2023) reported that 38% of participants resumed MA use within an observational period of 12 months, however, the patients in their study were still engaged in a treatment program with monthly sessions, meaning that this was not a post-treatment follow-up^20^. In the context of pregnant and parenting women, Porowski et al. (2024) reported a recurrence rate of approximately 40% within a six-month follow-up period, counting any use of addictive substances, including alcohol but excluding tobacco^21^.

Taken together, a comparison with previous studies reveals a remarkably low rate of substance use recurrence in our sample. This is particularly noteworthy given our relatively long follow-up period. To gain insight into the factors that may contribute to the potential long-term effectiveness of treatment programs, we examined potential moderators of treatment success in comparable samples. Women with longer histories of MUD prior to admission were more likely to report post-treatment substance use. In line with this, a shorter duration of MA use before treatment increased the odds of a successful outcome at the 3-month follow-up after group psychotherapy was added to standard psychiatric care for MUD^22^. Furthermore, having a higher number of underage children and experiencing mother-child separation before MAMADAM were positively inter-correlated predictors of substance use recurrence after MAMADAM. These results align with other studies that have shown that women who consume MA often struggle with parenting stress^11,23–25^.

Mao et al. (2024) employed a probabilistic, dynamic mathematical model to demonstrate that a combination of stressors, a consumer-friendly environment, and joyful events increases the likelihood of relapse. This model provides a theoretical framework that corroborates our finding that increased recurrence rates are associated with psychosocial stressors and environmental triggers. Conversely, exposure to a mild, yet continuous, source of contentment acts as a protective factor, in addition to the known factors such as being embedded in an addiction support system^16^. MAMADAM aims to mitigate socio-economic stressors, thereby helping patients disengage from a consumption-oriented environment and circumvent high-risk scenarios, such as excessive partying. Its specific components, including its MA-specific group psychotherapy^22,26^, have proven effective in engaging expecting and new parents in treatment, helping them recover and maintain the custody of their children^9,10,12,13,27^. Keeping families together and enabling the experience of parenthood are at the core of MAMADAM, creating a sense of contentment that is considered a protective factor.

A comparison with the results from Huang et al. (2023), in which the majority of recurrences took place within the first three months following treatment initiation, also suggests the following explanation for the sustainability of MAMADAM: In this period of heightened vulnerability during early abstinence, our patients remain subject to considerable external monitoring and may be driven by a stronger motivation than later on due to fear of losing custody of their children in the event of substance use. At this juncture, the therapeutic approach is highly structured, comprising unannounced random drug tests and weekly outpatient appointments. Another potential explanation is the flexibility of the MAMADAM program. As evidenced by the aforementioned study by Huang, comorbid disorders and a longer duration of substance use prior to treatment initiation have also been identified as complicating factors^20^. This was also evident in our sample. The structure of our psychiatric department enabled us to adapt the duration and intensity of therapy flexibly. In addition to addiction-focused treatment, our facility was able to treat comorbidities in a disorder-specific manner^10,12^.

### Limitations

The relatively high abstinence rate in the 39 women with follow-up data should be interpreted cautiously.

First, abstinence rates were likely lower among the 75 women for whom we could not collect follow-up data, as most were not in stable abstinence at MAMADAM discharge. A response rate of 34% at a follow-up period of about 4.5 years on average is rather high in comparison to, for example, Kamp et al. (2020)^19^, who reported a follow-up rate of approximately 23% after 18 months, while Brecht et al. (2014) reported a follow-up rate of 79% after 3 years^17^.

Second, substance recurrence was not biochemically verified but only based on retrospective self-reports in our study. Even though anonymity was assured, some women may not have reported relapse, for example due to fear of stigma or consequences such as loss of child custody. Studies indicated that the actual number of abstinence violations may be higher if unannounced drug tests are used^28^. However, other studies found self-reports of drug use to be reliable^29,30^.

Third, abstinence rates may be influenced by different patterns of polysubstance use history, even though our groups did not significantly differ in the prevalence of substance use comorbidity.

Finally, our MAMADAM care concept and some of our findings may not be easily transferable to patient populations in healthcare systems with different social, financial, or legal contexts.

## Conclusions and perspectives

Our findings indicate that the MAMADAM treatment program is not only efficacious but also sustainable. This highlights the potential of treatment programs that utilize pregnancy and parenthood as catalysts for behavior change, which can bring about long-term recovery from SUD. Based on many years of involvement in the clinical practice of MAMADAM and with due consideration of the data available, we consider the flexible adaptation of therapeutic resources to individual needs and comorbidities to be an essential factor in the program’s success.

Yet, women who reported substance use after MAMADAM were more likely to have more minor children, live apart from at least one minor, and have a longer history of MA use at the time of admission to MAMADAM. These associations indicate the importance of parenting in sustaining abstinence within the complex dynamics of socioeconomic and sociolegal stressors associated with MUD. Future research should investigate whether stronger partnerships with government and community agencies could be beneficial in helping these women maintain long-term abstinence. Targeting interpersonal and parenting skills to build supportive relationships between patients and their families may ultimately direct MA-specific integrated care toward the recovery and resilience of the whole family.

**Table 1:** Patient characteristics stratified by availability of follow-up.

	Follow-up available?		Group differences
	No (*N* = 75)	Yes (*N* = 39)	
**After MAMADAM treatment**			
Days from discharge to follow-up	N/A	1640 ± 381	N/A
Days of abstinence from discharge to follow-up	N/A	1089 ± 683	N/A
**MAMADAM treatment**			
Duration in days	275 ± 428	277 ± 291	U = 1587.00, z = 0.74, *p* = 0.46^A^
Stable abstinence at discharge	44%	74%	Χ^2^ (1) = 9.53, *p* = 0.002^C^*
**Baseline at admission to MAMADAM**			
Years of age	28 ± 6	28 ± 5	U = 1497.00, z = 0.21, *p* = 0.84^A^
Childless	8%	5%	Χ^2^ (1) = 0.32, *p* = 0.71^F^
Underage children	2 ± 1	2 ± 1	U = 1495.00, z = 0.20, *p* = 0.84^A^
Mother-child separation (*N* = 106 mothers)	62%	59%	Χ^2^ (1) = 0.08, *p* = 0.77^C^
Single	53%	54%	Χ^2^ (1) = 0.00, *p* = 0.96^C^
Stable housing	75%	87%	Χ^2^ (1) = 2.42, *p* = 0.12^C^
Employed	9%	15%	Χ^2^ (1) = 0.93, *p* = 0.36^F^
Criminal record (*N* = 109)	45%	37%	Χ^2^ (1) = 0.54, *p* = 0.46^C^
Years of methamphetamine use (*N* = 108)	6 ± 5	8 ± 7	U = 1557.50, z = 1.36, *p* = 0.17^A^
Substance use comorbidity^T^	47%	62%	Χ^2^ (1) = 2.27, *p* = 0.13^C^
Prior withdrawal program	52%	51%	Χ^2^ (1) = 0.01, *p* = 0.94^C^
Partner with substance use disorder^T^ (*N* = 98)	58%	45%	Χ^2^ (1) = 1.49, *p* = 0.22^C^

Statistical analyses were performed on data from all women (*N* = 114) unless involving variables with reduced sample sizes due to missing values. Results are presented as group M ± SD or percentages of women within the respective group of follow-up availability (yes or no). Housing was considered stable (own apartment, condominium, or house) or unstable (supported transitional accommodation with children, living in others’ homes, or homelessness). Mother-child separation meant mothers living apart from at least one of their underage children.

T (excluding tobacco use disorder).

A (asymptotic significance of Mann-Whitney U test).

C (Pearson’s chi-square test).

F (Fisher’s exact test).

* (significant at *p* < 0.05).

**Table 2:** Patient characteristics stratified by sustained abstinence at follow-up.

	Abstinent^T^ since discharge?		Group differences
	No (*N* = 23)	Yes (*N* = 16)	
**After MAMADAM treatment**			
Days from discharge to follow-up	1605 ± 337	1689 ± 444	U = 191.00, z = 0.20, *p* = 0.85^E^
Days of abstinence from discharge to follow-up	672 ± 476	1689 ± 444	U = 344.00, z = 4.58, *p* < 0.001^E^*
**MAMADAM treatment**			
Duration in days	272 ± 265	285 ± 333	U = 176.00, z = −0.23, *p* = 0.83^E^
Stable abstinence at discharge	70%	81%	Χ^2^ (1) = 0.68, *p* = 0.48^F^
**Baseline at admission to MAMADAM**			
Years of age	30 ± 5	27 ± 5	U = 122.00, z = −1.78, *p* = 0.08^E^
Childless	0%	13%	Χ^2^ (1) = 3.03, *p* = 0.16^F^
Underage children	2 ± 1	2 ± 1	U = 108.00, z = −2.25, *p* = 0.03^E^*^#^
Mother-child separation (*N* = 37 mothers)	74%	36%	Χ^2^ (1) = 5.27, *p* = 0.02^C^*
Single	57%	50%	Χ^2^ (1) = 0.16, *p* = 0.69^C^
Stable housing	87%	88%	Χ^2^ (1) = 0.00, *p* = 1.00^F^
Employed	13%	19%	Χ^2^ (1) = 0.24, *p* = 0.67^F^
Criminal record (*N* = 35)	43%	29%	Χ^2^ (1) = 0.73, *p* = 0.39^C^
Years of methamphetamine use (*N* = 39)	10 ± 7	5 ± 3	U = 90.50, z = −2.68, *p* = 0.01^E^*
Substance use comorbidity^T^	70%	50%	Χ^2^ (1) = 1.53, *p* = 0.22^C^
Prior withdrawal program	43%	63%	Χ^2^ (1) = 1.37, *p* = 0.24^C^
Partner with substance use disorder^T^ (*N* = 33)	52%	33%	Χ^2^ (1) = 1.12, *p* = 0.29^C^

Statistical analyses were performed on data from all women with follow-up data (*N* = 39) unless involving variables with reduced sample sizes due to missing values. Results are presented as group M ± SD or percentages of women within the respective group of abstinence maintenance (yes or no). Housing was considered stable (own apartment, condominium, or house) or unstable (supported transitional accommodation with children, living in others’ homes, or homelessness). Mother-child separation meant mothers living apart from at least one of their underage children.

T (excluding tobacco use disorder).

E (exact significance of Mann-Whitney U test).

C (Pearson’s chi-square test).

F (Fisher’s exact test).

* (significant at *p* < 0.05).

# (Despite the statistical difference in the distributions of values, the similarity in means and standard deviations suggests limited practical relevance.).

**Table 3:** Correlation between correlates of sustained abstinence at follow-up.

	Mother-child separation	Years of methamphetamine use
Number of children	*N* = 37, rs = 0.77, *p* < 0.001*	*N* = 39, rs = 0.38, *p* = 0.02*
Mother-child separation (0 = no, 1 = yes)	N/A	*N* = 37, rs = 0.41, *p* = 0.01*

Spearman’s Correlation (rs) between variables that were significantly related to sustained abstinence at follow-up (Table 2). * (significant at *p* < 0.05).

**Table 4:** Cox regression predicting the duration of abstinence after MAMADAM.

Covariates	Exp(B) with 95% CI	*p*
Years of methamphetamine use	1.09 (1.03–1.16)	< 0.001*
Days of MAMADAM treatment	1.00 (1.00–1.00)	0.66

Model	Χ^2^ (2)	*p*
Overall	8.72	0.01*
Change after simultaneously entering the two covariates	7.60	0.02*

Correlation	rs	*p*
Between the two covariates	0.07	0.69

Cox regression tested the predictive value of the variable with the strongest significance related to sustained abstinence at follow-up (i.e., years of methamphetamine use, Table 2) on the duration of sustained abstinence since discharge from MAMADAM, controlling for the duration of MAMADAM. *N* = 39. rs (Spearman’s Correlation). * (significant at *p* < 0.05).

The duration of methamphetamine use was displayed as a binary variable (20 patients with less than 7 years and 19 patients with ≥ 7 years) to illustrate its effect as a continuous variable (*N* = 39) in the Cox regression (Table 4).

## Data Availability

The datasets generated and analyzed during the current study are available from the corresponding author on reasonable request.
